# Sir James Crichton Browne on Physical Education

**Published:** 1904-10-15

**Authors:** 


The Hospital.

A Journal of

The Medical Sciences and Hospital Administration

Vol. XXXVII.—No. 941. OCTOBER 15, 1904.

### Sir James Crichton Browne on Physical Education.

Sir James Crichton Browne, speaking as president for the year of the Salt Schools at Shipley last week, laid down with great precision some of the leading principles by which, he thinks, physical education should be governed. Like all good teachers, he began at the beginning, and dwelt upon the importance of the muscular and sensory activities which may be excited in the infant by the "dandling and tossing" of the mother, and which may remain dormant, with harmful or even fatal consequences, in infants who are deprived of this wholesome and natural stimulus, and who are only taken from their cots to be fed and replaced. While admitting that the labour of women in factories entails of necessity the abandonment by those of them who are mothers of the form of nursing which he so strongly recommends, he supplements this admission by the practical suggestion that the *crèche*, where it is a necessity, might be utilised as a training school in which girls could be taught the management of infants, and in which the infants, consequently, might be "dandled and tossed" to their hearts' content. With the arrival of powers of independent locomotion physical education is, he thinks, sufficiently provided for by the active impulses of the child, and it is not until the age of seven has been attained that he would find scope for any useful interference with or guidance of these impulses on the part of the teacher. He would still leave country children to educate themselves physically by rural games and sports, but for town children, whose only playgrounds are streets, courts, and alleys, he admits the necessity, or at least the value, of systematic guidance from adults. He points out that the training of the muscles involves the growth of the cerebral motor centres with which the several groups are associated in action, and that the growth of these centres involves that of the brain considered as a whole, and in relation to all the functions which its several parts are ultimately to fulfil. He also lays stress upon the physiological fact that the several motor centres tend towards development in a stated order and at stated times, with the general result that lost time cannot be regained in relation to them. If the hand, for example, be not trained at a comparatively early period of life, its motor centres and its muscles will alike have missed their chance and it can never afterwards be brought to the highest degree of dexterity He draws a piteous picture of boys and girls of 15, intended ultimately for manual industries, but suffered to sit at desks poring over decimals or geography, while their hands are becoming flaccid and their fingers stiff. He teaches the two-fold lesson, first, that the brain can never grow to its highest attainable perfection independently of the muscular system; and secondly, that the muscular system can never be well developed independently of regular and progressive exercise. To compel children to be clumsy is also to train them to be stupid.

Sir James discussed in detail, at considerable length, some of the recommendations which have been made with regard to physical education, and expressed his dissent from those which demand interference with the performance of a natural function, such as breathing. He thought it unnecessary that children should be taught to breathe through the nostrils only, and maintained that they cannot do so under the stress of active exertion. We have no wish to advocate superfluous teaching of any sort, but we think, with great submission, that Sir James is in error. We do not know how he will receive our criticism. Victor Hugo introduced an Englishman into one of his novels, and called him Jonjimjak. An actual Englishman, in an interview with the great writer, assured him that Jonjimjak was not an English name. "You may be an Englishman," was the reply, "but I am Victor Hugo." In like manner, Sir James may be inclined to assert his superior authority. But the fact remains that, among the nearly extinct North American aborigines, at a time when they were capable of extraordinary physical exertion, as hunters or warriors, the precept to "shut your mouth" was the foundation of all other education, and was enforced upon the young by the most severe discipline. Catlin founded upon his experiences among the American tribes a curious book upon the subject, still sometimes to be found in catalogues, and abundantly illustrated by his skilful pencil. After depicting open-mouthed men and boys in every variety of ugliness and stupidity, he says that he refrains from giving illustrations of the fairer sex, and would only remind them, while counselling them to be careful about closure of the mouth at night, that "idiots asleep cannot be angels awake." Sir James declares that not all nostrils are sufficiently wide to permit of breathing being conducted through them to the exclusion of the mouth; but it is certain that nothing would tend more than breathing through them to promote their growth and development. It is also certain that the open mouth affords a much more easy passage to the microbes of tubercle or of diphtheria than the comparatively narrow, winding, and hair-lined nostrils; and it cannot be doubted that the general enforcement of nasal breathing and of shut mouths in schools would lead to the prompt detection of the cases in which, by reason of the presence of adenoids or other obstructions, the practice could not be pursued. Sir James is also in error, we think, in his contention that it is not possible to breath only through the nostrils in public speaking or in reading aloud. To show how this can be and should be done, and how the tongue may be so placed during inhalation as to check the entrance of air through the mouth, even if the lips be parted, forms the foundation of the teaching of most elocution masters, and the practice affords an effectual protection against that dryness of the mouth during speaking which forms the great trouble of the untaught and unskilled orator, and which he vainly seeks to remedy by frequent sips of water. In one sense "shut your mouth" is the last advice which we should desire to offer to Sir James, but we should be glad to see the abandonment of his opposition to that advice when it is offered to the public in general.

